# Myoepithelioma of the Accessory Parotid Gland: A Case Report

**DOI:** 10.7759/cureus.115358

**Published:** 2026-08-28

**Authors:** Jagadiswar Pati

**Affiliations:** 1 Surgery, Tata Main Hospital, Ramgarh, IND

**Keywords:** accessory parotid gland, mid-cheek mass, myoepithelioma, salivary gland neoplasm, superficial parotidectomy

## Abstract

Myoepithelioma is an extremely rare benign tumor of the salivary glands that poses a diagnostic challenge because its clinical, radiologic, and cytopathologic characteristics may resemble those of several other salivary gland tumors. Accessory parotid myoepithelioma is even rarer. A 24-year-old female patient presented with a five-year history of a gradually increasing, painless swelling in the right mid-cheek region. Clinical examination revealed a firm, well-demarcated, nontender swelling in the right mid-cheek region without evidence of facial nerve involvement. Ultrasonographic evaluation revealed a multilocular mass superior to the right parotid gland, and fine-needle aspiration cytology was suggestive of pleomorphic adenoma. The patient underwent superficial parotidectomy with complete excision of the tumor while preserving the facial nerve branches. Histopathological examination confirmed the diagnosis of myoepithelioma, demonstrating sheets and cords of myoepithelial cells within a collagenous stroma. The postoperative period was uneventful, with no recurrence or facial nerve dysfunction during the 2.5 years of follow-up. This report highlights the importance of including accessory parotid tumors in the differential diagnosis of cheek masses. Histopathological examination is essential for establishing a definitive diagnosis, and complete surgical excision remains the optimal management strategy.

## Introduction

Neoplasms of the salivary glands are rare in general, and of all types of tumors, parotid gland tumors are the most common, comprising 70-80% of all salivary gland tumors, 2-3% of all head and neck tumors, and 0.6% of all tumors [1]. Within the parotid gland, the great majority of tumors arise from the main superficial or deep lobes; the accessory parotid lobe, a discrete island of glandular tissue lying anterior to the main gland along the parotid duct, gives rise to only about 1-8% of all parotid neoplasms [2]. Because accessory parotid tumors present as isolated mid-cheek masses rather than preauricular swellings, they are frequently overlooked or mistaken for cutaneous, subcutaneous, or mimetic muscle lesions, and several surgical series report a higher relative proportion of malignancy in this location than in the main gland. The close anatomical relationship of accessory parotid lesions to the parotid duct and facial nerve branches may also present additional challenges during surgical planning and excision [2,3].

Myoepithelioma was formally distinguished from pleomorphic adenoma in the World Health Organization classification of salivary gland tumors based on its composition, as it is composed almost entirely of neoplastic myoepithelial cells with little or no ductal or chondromyxoid component. It remains a rare tumor, accounting for approximately 1-1.5% of all salivary gland neoplasms in most large series, with the parotid gland affected in 40-90% of reported cases and the minor salivary glands, particularly the palate, accounting for most of the remainder [4,5]. Myoepithelioma arising specifically from the accessory lobe of the parotid gland is exceedingly rare, with only a small number of cases reported in the literature [6].

The clinical presentation of accessory parotid tumors is nonspecific, and the histopathologic similarities between myoepithelioma and pleomorphic adenoma make preoperative diagnosis challenging; in fact, the former is frequently indistinguishable from the latter on fine-needle aspiration cytology [7]. The following case report presents a rare case of myoepithelioma involving the accessory parotid gland and emphasizes the significance of including this entity in the differential diagnosis of lesions of the mid-cheek region.

## Case presentation

A 24-year-old female presented to the surgical outpatient department with a complaint of painless swelling over the right cheek for five years. The mass had gradually increased in size over time, and there were no factors that aggravated or relieved the symptoms. There was no history of trauma or infection preceding the onset of the mass. She had no significant past medical or surgical history, history of substance abuse, or exposure to therapeutic or occupational radiation. Upon examination, there was a firm, well-demarcated, nontender swelling measuring approximately 3 × 1 × 1 cm, palpable over the right mid-cheek region below the zygomatic arch. There was no cervical lymphadenopathy; the overlying skin was normal, and facial nerve function was intact on clinical examination. The possible clinical diagnoses were pleomorphic adenoma, adenoid cystic carcinoma, and a mesenchymal soft-tissue tumor.

**Figure 1:**
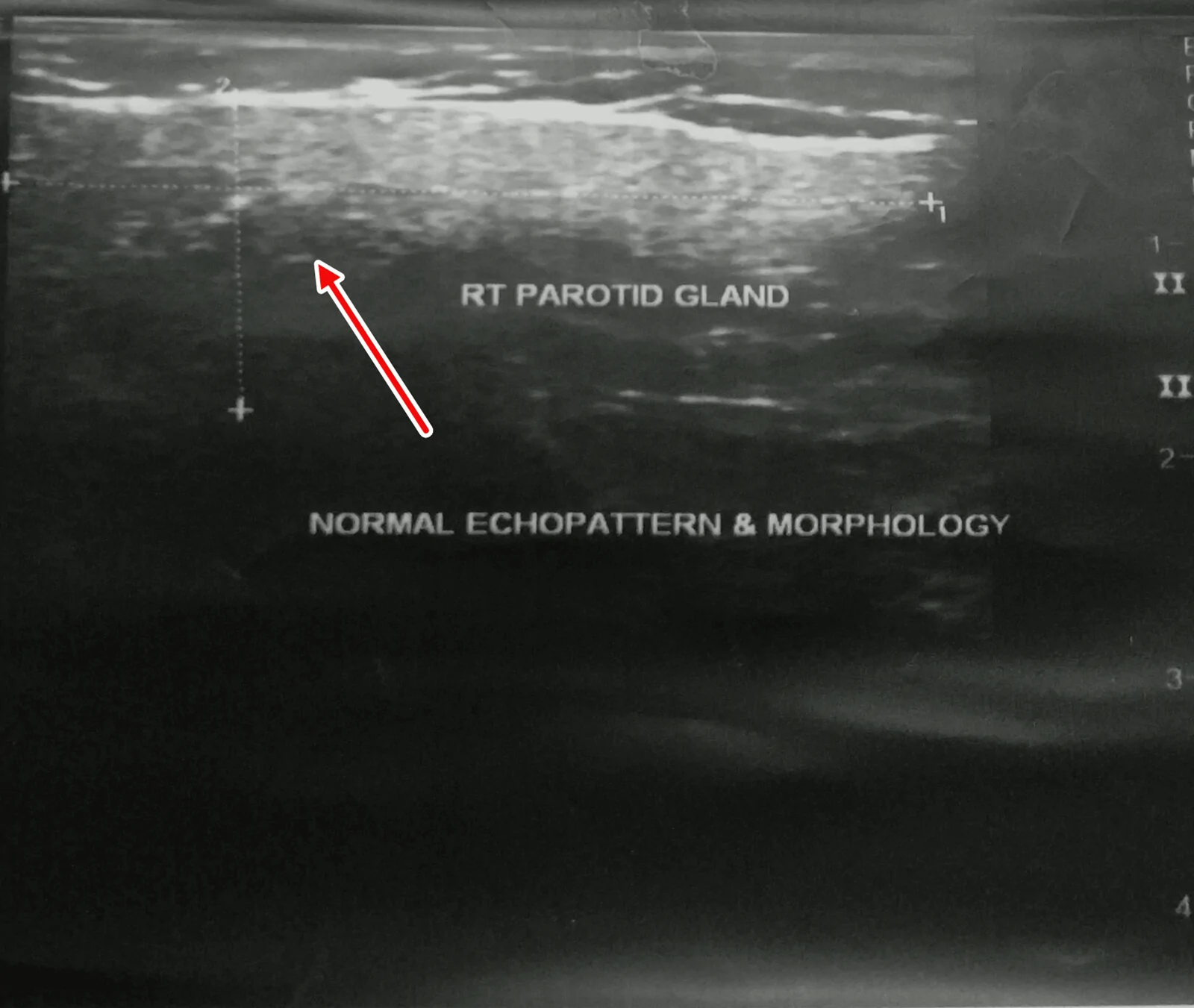
High-resolution ultrasonography showing a multilobulated mass superior to the right parotid gland

**Figure 2:**
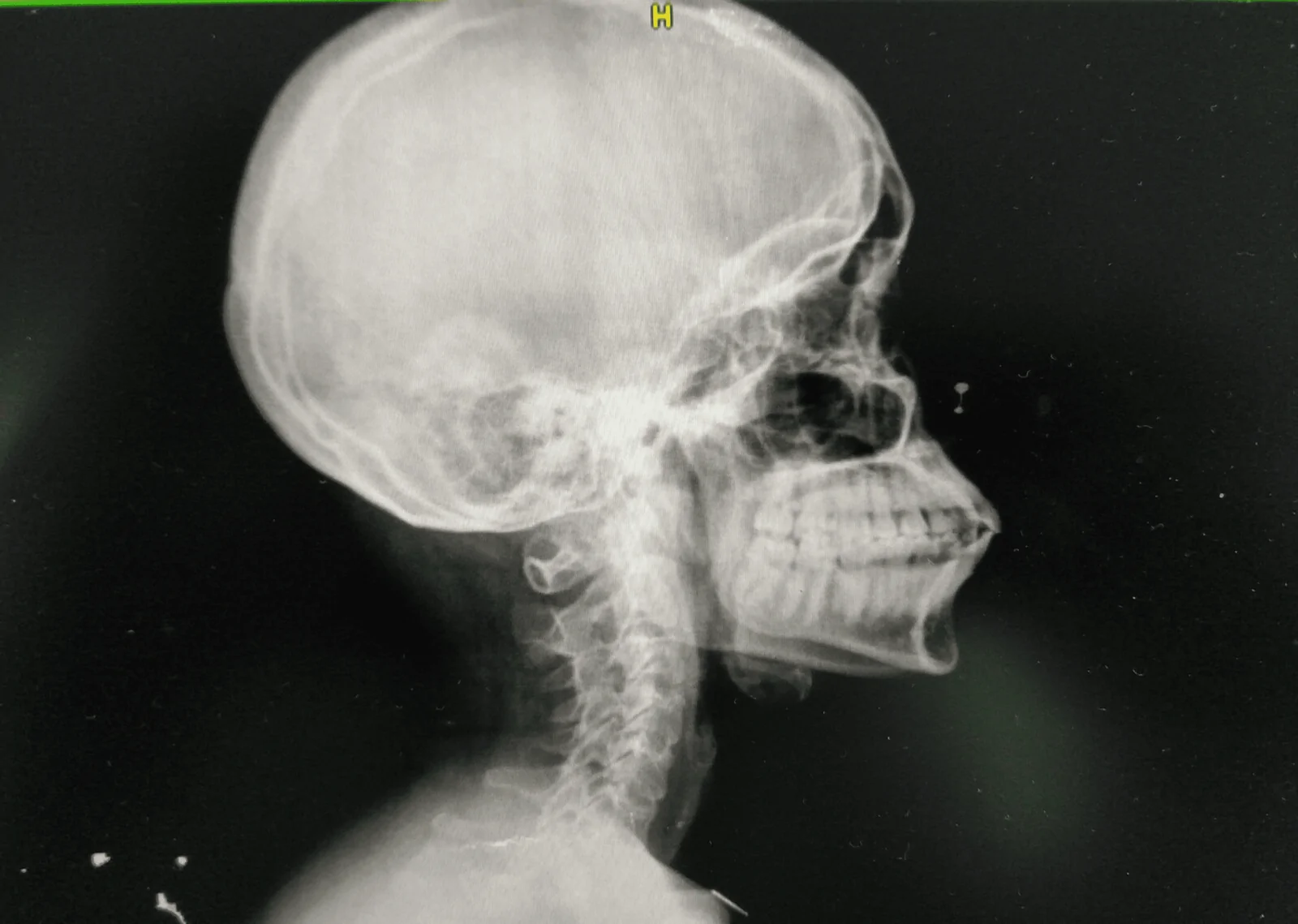
Plain lateral skull and mandible radiograph showing no evidence of bony involvement

The routine hemogram was normal. High-resolution ultrasonography of the right parotid area (Figure 1) showed a multilobulated mass superior to the right parotid gland, without any evidence of cervical lymphadenopathy. A plain lateral skull and mandible radiograph (Figure 2) revealed no signs of bony erosion or involvement of the mandible or skull base.

Fine-needle aspiration cytology of the right parotid area revealed a highly cellular smear comprising predominantly myoepithelial cells with an epithelial cell component, arranged in fibrillary stromal fragments with areas of pseudotubular arrangement. The cells were oval, polygonal, or spindle-shaped, with round to oval nuclei showing bland nuclear features and variable amounts of cytoplasm. No cytologic atypia was identified. The findings were suggestive of pleomorphic adenoma, and histopathological examination was recommended for confirmation.

**Figure 3:**
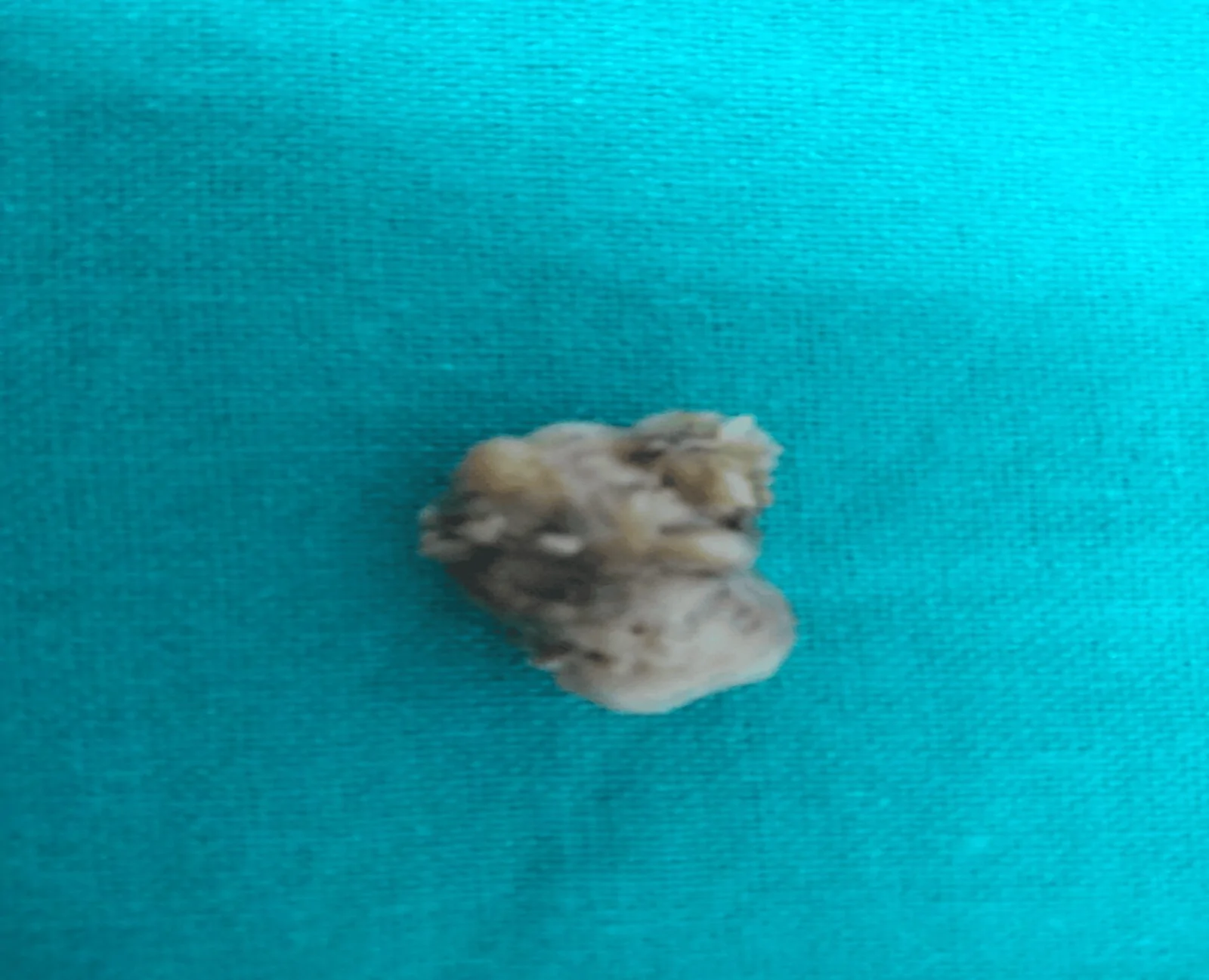
Gross surgical specimen

Under general anesthesia, the patient underwent right superficial parotidectomy with complete excision of the accessory parotid mass. A vertical incision was made directly over the lesion, parallel to the tragal crease. A well-encapsulated, lobulated mass measuring 3 × 1 × 1 cm was identified superior to the parotid duct, with loose adhesions to the rest of the parotid gland, and was completely excised. The facial nerve was dissected, and its branches were carefully preserved. Grossly, the excised specimen was a well-encapsulated, lobulated, tan-brown, firm tumor measuring 2 × 1.2 × 0.8 cm (Figure 3).

Histopathological examination confirmed the diagnosis of benign myoepithelioma. Microscopy revealed a fairly circumscribed, partly encapsulated tumor composed of sheets and cords of myoepithelial cells within a collagenous stroma. The tumor cells showed eosinophilic cytoplasm and round nuclei with dispersed chromatin, with mild nuclear pleomorphism and occasional mitotic figures. There was no evidence of perineural or lymphovascular invasion. These findings were suggestive of a benign myoepithelial tumor (myoepithelioma).

The immediate postoperative period was uneventful. There was no facial nerve weakness or paralysis, and no hematoma or seroma was noted. The surgical wound healed well. The patient underwent regular outpatient clinical follow-up for 2.5 years after surgery, with assessments for local recurrence and facial nerve dysfunction. No evidence of local recurrence or facial nerve dysfunction was observed during the follow-up period. Table 1 summarizes the patient's presentation, diagnosis, management, and follow-up.

**Table 1:** Timeline of clinical events, diagnosis, treatment, and follow-up FNAC: fine-needle aspiration cytology

Timeline	Clinical event
5 years before presentation	Patient noticed a painless swelling over the right mid-cheek that gradually increased in size over time
Initial presentation	Presented to the surgery outpatient department for evaluation of the persistent swelling
Clinical examination	A firm, well-defined, nontender mass, about 3 × 1 × 1 cm, was found below the zygomatic arch. There was no cervical lymphadenopathy, and facial nerve function was normal
Diagnostic workup	Ultrasonography revealed a multilobulated mass superior to the right parotid gland. Skull radiography showed no bony involvement. FNAC suggested cellular pleomorphic adenoma
Surgical management	Right superficial parotidectomy with complete excision of the mass and preservation of facial nerve branches
Histopathological diagnosis	Histopathological examination confirmed myoepithelioma of the accessory parotid gland
Postoperative outcome	Uneventful recovery without facial nerve weakness or wound-related complications
Follow-up	At 2.5-year follow-up, there was no evidence of local recurrence or facial nerve dysfunction.

## Discussion

The accessory parotid gland is a separate lobule of salivary tissue lying superficial to the masseter muscle and superior to the parotid duct and is usually connected to the parotid duct by a short duct called the accessory duct. It has been found in more than half of cadaveric dissections, but its reported prevalence varies from approximately 21-69% of specimens examined, depending on the method of dissection [8]. It is located separately from the main gland and, when excised, appears as a solitary mass in the middle of the face, rather than the more familiar preauricular or infra-auricular mass of a typical parotid tumour. This atypical location often leads to delays in diagnosis and misclassification as a cutaneous or mimetic-muscle tumour [2,3]. Pooled data indicate that accessory lobe tumors account for 1-8% of all parotid gland tumors but have a significantly higher risk of malignancy than tumors arising in the main parotid gland [2].

Myoepithelioma itself is a rare tumor, comprising about 1-1.5% of all salivary gland tumors, with the parotid gland being the most common site of myoepithelioma [9]. It is recognized as a distinct lesion because of the presence of a predominantly myoepithelial neoplasm without ductal or chondromyxoid stroma, a histological distinction that cannot be established solely by means of cytology and imaging [4,10]. The diagnostic overlap is perfectly demonstrated in our patient, who had a fine-needle aspiration cytology report suggestive of pleomorphic adenoma but whose diagnosis remained inconclusive until histopathological examination, as described by Das et al. in a parotid myoepithelioma that was only diagnosed post-excision [11]. Radiologically, MRI is considered superior to CT for characterizing salivary gland tumors owing to its better soft-tissue contrast, but the appearances of myoepithelioma frequently mimic those of pleomorphic adenoma, and imaging alone cannot substitute for histopathological confirmation [12,13].

Cases of myoepithelioma originating from the accessory parotid gland are quite rare. One such case was reported by Kawashima et al., in which the tumor was detected following the removal of a mass on the cheek [14]. Iguchi et al. later described the pathological and MRI findings of an epithelioid variant of accessory parotid myoepithelioma, highlighting the importance of MRI in surgical planning, despite its low specificity [12]. Luksic et al. conducted a study of accessory parotid gland tumors from a single center over 32 years and emphasized once again that, regardless of the histopathological type, complete excision with facial nerve preservation is the key factor for success [15]. Accessory parotid tumor cases, including salivary duct papillomas and other rare variants, also support this concept of total excision based on imaging and pathology [13].

Even though myoepitheliomas generally exhibit benign behavior, cases have been described in the literature in which the tumor has undergone malignant transformation into myoepithelial carcinoma, especially when there is incomplete removal or recurrence, emphasizing the need for complete removal of the lesion with an intact capsule during the primary surgery [4,10]. Myoepithelial neoplasms have also rarely been reported at other unusual head and neck sites, such as the vallecula, reflecting the broad distribution of myoepithelial cells across the secretory tissues of the upper aerodigestive tract [16]. In our patient, the tumor was well encapsulated with only minimal adhesion to the main gland, permitting complete excision through a superficial parotidectomy approach with preservation of all facial nerve branches, consistent with outcomes reported in comparable cases of parotid and accessory parotid myoepithelioma treated by superficial parotidectomy [17,18]. A limitation of this report is the unavailability of histopathological images for inclusion; however, the relevant histopathological findings have been described in detail in the manuscript.

## Conclusions

A slowly enlarging, painless mid-cheek mass should prompt consideration of an accessory parotid gland tumor. Accessory parotid myoepithelioma is an extremely rare benign salivary gland neoplasm and may be difficult to distinguish from pleomorphic adenoma on preoperative evaluation. Histopathological examination remains essential for establishing a definitive diagnosis. Complete surgical excision with preservation of the facial nerve is an effective treatment approach, with favorable clinical outcomes during follow-up.
